# The role of animal-assisted therapy in the management of people with dementia: a systematic literature review

**DOI:** 10.1192/bjo.2021.734

**Published:** 2021-06-18

**Authors:** Syed Nabeel Javaid

**Affiliations:** LYPFT NHSFT

## Abstract

**Aims:**

The aim of this systematic literature review was to determine the evidence-based effectiveness of animal assisted interventions and to look at the factors that limit implementation of this intervention.

**Background:**

Dementia is a major health issue worldwide impacting not only on the people diagnosed with dementia, but also on their families and caregivers, and the healthcare professionals. The symptoms of dementia include cognitive impairment that can range from mild to severe, and behavioural and psychological symptoms which have debilitating effects on functional capacity and quality of life. A number of non-pharmacological interventions are being developed to help people with dementia. Animal assisted therapy is one of those interventions that has demonstrated positive effects on various aspects of dementia (Filan and Llewellyn-Jones, 2006). However, there are limitations to its use and feasibility of animal assisted therapy programmes is unclear.

**Method:**

Only randomised-controlled trials (RCTs) were to be included to evaluate high quality evidence. A systematic literature search was carried out to find using the PubMed and Cochrane databases and a search of the NICE website. Literature was screened according to inclusion and exclusion criteria. Eight randomised-controlled trials were selected to be used in this systematic review to assess the effectiveness of animal-assisted therapy.

**Result:**

The results regarding the effectiveness of animal assisted therapy were variable. There was some improvement demonstrated in symptoms of depression, agitation, behaviour and cognitive impairment. Quality of life and activities of daily living also demonstrated positive outcomes. There was a reduction in the risk of falls in people with dementia. However, the studies conducted demonstrated limited methodologies. The factors limiting the use of animal assisted therapy were found to be concerns around adverse events to animals, issues of animal welfare and economic feasibility of animal assisted therapy programmes.

**Conclusion:**

Further research needs to be done using properly conducted randomised controlled trials with larger sample sizes to formally assess people's perceptions regarding therapy animals and develop clear guidelines and protocols for integrating these interventions in healthcare.

